# Antibacterial performance of biomimetic hydroxyapatite–functionalized titanium surfaces against *Staphylococcus aureus* and *Escherichia coli*: an *in vitro* study

**DOI:** 10.3389/froh.2026.1850878

**Published:** 2026-06-18

**Authors:** Adel S. Alagl, Marwa Madi

**Affiliations:** Department of Preventive Dental Sciences, College of Dentistry, Imam Abdulrahman Bin Faisal University, Dammam, Saudi Arabia

**Keywords:** *Escherichia coli*, functionalized, hydroxyapatite, *Staphylococcus aureus*, titanium

## Abstract

**Background:**

Peri-implantitis, driven by bacterial biofilm formation on implant surfaces, remains a leading cause of dental implant failure. Surface modification strategies that combine antibacterial properties with biocompatibility are therefore of considerable clinical interest. This *in vitro* study aimed to evaluate the antibacterial performance of hydroxyapatite (HA)-coated titanium surfaces, produced by radiofrequency (RF) magnetron sputtering with hydrothermal post-treatment, against *Staphylococcus aureus* and *Escherichia coli* in single-species and dual-species biofilm models.

**Methods:**

Commercially available grade 2 titanium discs were divided into HA-coated (Ti-HA) and uncoated (Ti) groups (*n* = 8 per group per condition). Biofilm formation was assessed after 24 h of incubation at 37 °C using colony-forming unit (CFU) counts on selective media and optical density (OD) measurements at 630 nm. Statistical analysis included two-way ANOVA on log-transformed CFU data, Mann- Whitney *U* tests for pairwise comparisons, and Kruskal-Wallis tests for within-surface species comparisons.

**Results:**

Ti-HA surfaces demonstrated substantial antibacterial efficacy across all conditions, with CFU reductions ranging from 97.9% to 99.7% against both *S. aureus* and *E. coli* in single and dual species biofilm models (all *p* < 0.001). Surface type was the dominant determinant of bacterial viability, accounting for 74.1% of total variance (*η*2 = 0.741). Biofilm optical density measurements corroborated these findings across all conditions. The antibacterial effect was species-dependent, with greater efficacy against Gram-positive *S. aureus *than Gram-negative *E. coli*, while the dual species environment did not significantly alter coating performance for either species.

**Conclusions:**

These findings provide proof-of-concept evidence that sputtered HA-coated titanium surfaces offer potent antibacterial activity against single and polymicrobial biofilms. While supporting the potential of this dual-function strategy to combine antibacterial protection with osteoconductivity, clinical translation requires further validation under simulated oral conditions that includes salivary pellicle formation and physiological loading as well as comprehensive *in vivo *studies.

## 1 Introduction

Titanium and its alloys remain the most widely used materials for dental and medical implants due to their excellent biocompatibility, mechanical strength, and corrosion resistance (1, 2). The long-term success of dental implants is primarily determined by the biological response at the tissue–implant interface (3). Despite their generally high success rates, approximately 5%–10% of dental implants still fail, mainly due to impaired osseointegration or biological complications (4). Among these, peri-implantitis a biofilm-induced inflammatory disease remains the most frequent cause of implant failure (5).

The formation of microbial biofilms on implant surfaces plays a key role in the initiation and persistence of peri-implantitis. Pathogenic bacteria colonize implant surfaces by forming biofilms that protect them from environmental stress and antimicrobial agents, thereby enhancing their virulence and resistance (6). Indeed, biofilm formation on implant surfaces has been identified as a primary etiological factor for peri-implantitis (7). The microorganisms most commonly associated with implant-related infections include *Staphylococcus aureus*, coagulase-negative staphylococci such as *Staphylococcus epidermidis*, *Escherichia coli*, *Pseudomonas aeruginosa*, and certain yeasts. Polymicrobial infections occur in approximately 10%–11% of cases, often proving highly resistant to antibiotic therapy and persisting until the implant is surgically removed (8, 9).

To prevent bacterial colonization, various surface modification strategies either physical or chemical, or both have been explored to render implant surfaces antibacterial (10). However, the effectiveness of these treatments in preventing biofilm adhesion is unclear, as they may also affect the physical-chemical properties of dental implants, such as surface roughness (11). Greater roughness can actually promote biofilm adhesion and, in turn, lead to the early onset of peri-implantitis (12). Previous study (13) showed that dual layered silver–HA nanocoating on implant surface inhibited 100% bacterial growth in the surrounding media. Additionally, micro-implants with hydroxyapatite nanoparticles (HA) showed promising antibacterial activity causing cell membrane damage for *Streptococcus salivarius*, *Streptococcus mutans*, *Enterococcus faecalis* and *Streptococcus sanguinis* (14)*.*

These include coatings incorporating antibiotics (15) or antiseptic agents such as chlorhexidine (16). However, such approaches often exhibit only short-term antimicrobial efficacy, and chlorhexidine has been reported to exert cytotoxic effects on human cells (17). Therefore, safer and more durable antimicrobial strategies are needed.

Studies have investigated the incorporation of metallic elements or nanoparticles to achieve antibacterial protection without compromising biocompatibility (18). The addition of zinc to magnesium alloy biodegradable implants has been shown to provide antibacterial effects while maintaining tissue compatibility (19). Similarly, composite nanocoatings embedding silver nanoparticles within a bioactive glass/polyetheretherketone (PEEK) composite matrix have achieved surfaces that are both antibacterial and biocompatible (20).

In orthopedic and dental implant research, the local delivery of bioactive or antimicrobial agents has emerged as a promising approach (13, 21). This method maximizes the therapeutic effect at the target site over prolonged periods while minimizing systemic toxicity and treatment costs. Among these materials, hydroxyapatite (HA) stands out due to its biomimetic composition, osteoconductivity, and strong bonding to bone tissue (22, 23). Its use as a coating material has demonstrated the ability to enhance bone integration and reduce bacterial colonization, making it an ideal candidate for next-generation implant coatings. The RF magnetron sputtering technique offers the ability to deposit thin coatings (0.5–3 µm) with strong adhesion, a dense microstructure, and precise control over elemental composition (21, 24, 25).

HA coatings resemble the mineral composition of natural bone and may provide surface characteristics that discourage early bacterial attachment while promoting favorable tissue integration (26). However, the antibacterial performance of HA coatings remains insufficiently characterized, particularly in comparison with unmodified titanium substrates and across different bacterial species and biofilm models. Understanding these differences is essential for developing implant surfaces that can reduce infection risk and improve long-term clinical outcomes. We hypothesized that Ti–HA surfaces would demonstrate significantly lower bacterial viability and biofilm accumulation than uncoated Ti surfaces, with differential effects between Gram-positive and Gram-negative species and between single and dual species biofilm models. Thus, the aim of this *in vitro* study was to evaluate and compare the antibacterial performance of biomimetic hydroxyapatite-coated titanium surfaces with unmodified titanium surfaces against *Staphylococcus aureus* and *Escherichia coli* in single-species and dual-species biofilm models.

## 2 Materials and methods

### 2.1 Specimen preparation

Commercially available grade 2 pure titanium (Ti) discs (10 mm × 10 mm × 2 mm) were used in this *in vitro* study. Ethical approval was obtained from the Institutional Review Board of Imam Abdulrahman Bin Faisal University (IRB-2026-02-0073).

Titanium discs were divided into two groups: uncoated Ti (control) and hydroxyapatite–coated Ti (Ti–HA). For the coated group, discs were first sandblasted and acid-etched, then subjected to RF sputtering using an SPF-410H chamber (ANELVA Corp., Kawasaki, Japan). “RF sputtering was performed using a hydroxyapatite ceramic target (Ca/P molar ratio 1.67) at a forward RF power of 100 W, Argon working pressure of 0.5 Pa, and a substrate-to-target distance of 80 mm. The deposition time was calibrated to yield a coating thickness of approximately 1.1 µm, confirmed by cross-sectional SEM. Hydrothermal post-treatment was carried out at 120 °C for 20 h in an aqueous electrolyte solution consisting of 0.1 M Ca(NO_3_)_2_ and 0.06 M NH_4_H_2_PO_4_ (pH 7.4 ± 0.1), as previously described by Ozeki et al. (27) (Yamahachi Dental Manufacturing Co., Gamagori, Japan). All specimens, both coated and uncoated, were sterilized by autoclaving at 121 °C for 28 min and individually packaged in sterile polypropylene bags until use.

### 2.2 Surface characterization

Surface morphology was examined using scanning electron microscopy (SEM; FEI Inspect S50, Brno, Czech Republic) at an accelerating voltage of 20 kV. Surface roughness (Ra) was measured using a non-contact optical profilometer (Contour GT-K1; Bruker Nano Inc., Tucson, AZ, USA). For each specimen, three measurements were obtained at equidistant points and averaged to calculate the mean Ra value. Each specimen was mounted on the automated *x*-*y* stage and scanned using white light interferometry with the following settings: 5× magnification, 1 mm × 1 mm field of view, 1× scan speed, and 0.1 mm/s stage speed.

### 2.3 Bacterial strains, culture conditions and biofilm formation

Two reference bacterial strains were used: *Staphylococcus aureus* ATCC 29213 and *Escherichia coli* ATCC 25218. Prior to experiments, bacterial cultures were grown in Brain Heart Infusion (BHI) broth (400 mL) for 24 h at 37 °C with agitation at 150 rpm. After overnight incubation, bacterial suspensions were adjusted to a turbidity equivalent to 0.5 McFarland standard (1.5 × 10^8^ CFU/mL), verified by measuring optical density at 630 nm using a microplate reader (ELISA plate reader BioTek, Winooski, VT, USA). Each experimental batch included uninoculated Ti and Ti-HA discs processed identically with sterile medium.

Early-stage bacterial adhesion and initial biofilm formation were assessed under three culture conditions: (1) single-species *S. aureus*, (2) single-species *E. coli*, and (3) dual-species (*S. aureus* + *E. coli*). For single-species biofilms, 200 µL of the standardized bacterial suspension (1.25 × 10^8^ cells/mL) was transferred onto each Ti or Ti-HA disc placed in a sterile well plate. For dual-species biofilms, 100 µL of each bacterial suspension (1.25 × 10^8^ cells/mL per species; total volume 200 µL) was added to each disc. Plates were incubated at 37 °C for 24 h with gentle agitation to allow biofilm formation. Following incubation, non-adherent cells were removed by gently rinsing each disc twice with sterile distilled water.

### 2.4 Quantification of viable biofilm cells (CFU analysis)

Each pre-washed disc was transferred to a sterile microcentrifuge tube containing 2 mL of sterile distilled water and sonicated for 10 min at 35 kHz to detach adherent bacteria. Following sonication, tubes were vortexed briefly to ensure complete dispersal of detached cells. The resulting suspensions were serially diluted (10-fold) and 200 µL aliquots were plated on appropriate agar media according to the culture condition.

For single-species *S. aureus* biofilms, suspensions were plated on blood agar (BA) for viable cell counting. For single-species *E. coli* biofilms, suspensions were plated on MacConkey agar (MCA) for selective quantification. For dual-species biofilms, suspensions were plated simultaneously on mannitol salt agar (MSA) for selective quantification of *S. aureus* and MacConkey agar (MCA) for selective quantification of *E. coli*, allowing independent enumeration of each species within the mixed biofilm.

**Figure 1:**
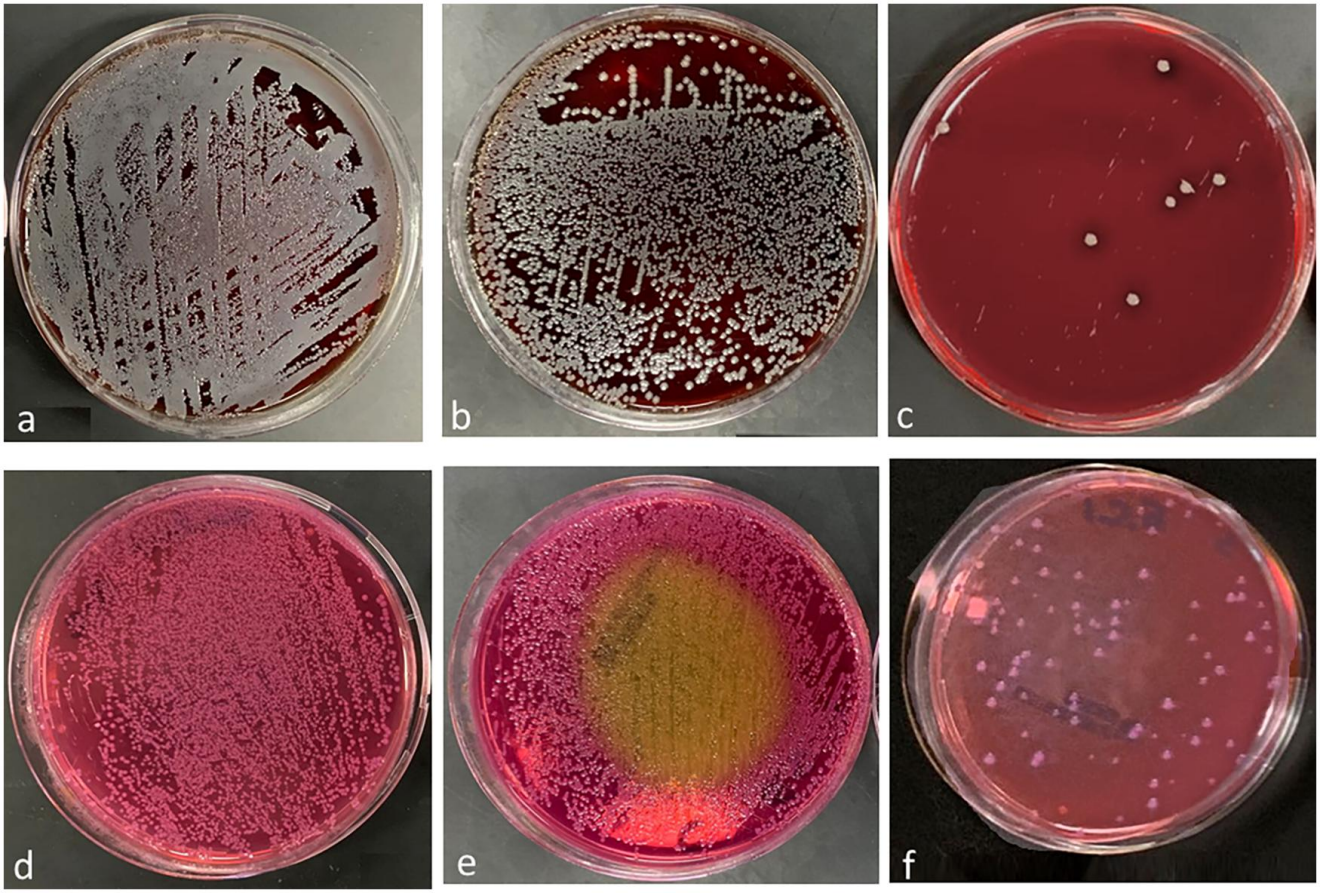
Representative agar plate images showing colony-forming unit (CFU) growth after 24 h of incubation at 37 °C. Upper row (*Staphylococcus aureus* on blood agar): **(a)** positive control (*S. aureus* culture without titanium disc), demonstrating characteristic hemolytic colony growth; **(b)** bacteria recovered from uncoated Ti disc, showing dense, confluent colony formation indicative of heavy bacterial colonization; **(c)** bacteria recovered from Ti–HA disc, showing markedly fewer colonies, reflecting the substantial antibacterial effect of the hydroxyapatite coating. Lower row (*Escherichia coli* on MacConkey agar): **(d)** positive control (*E. coli* culture without titanium disc), demonstrating typical lactose-fermenting colony growth; **(e)** bacteria recovered from uncoated Ti disc, showing dense colony formation with characteristic color change from lactose fermentation; **(f)** bacteria recovered from Ti–HA disc, showing visibly reduced colony counts compared to the uncoated surface.

**Figure 2:**
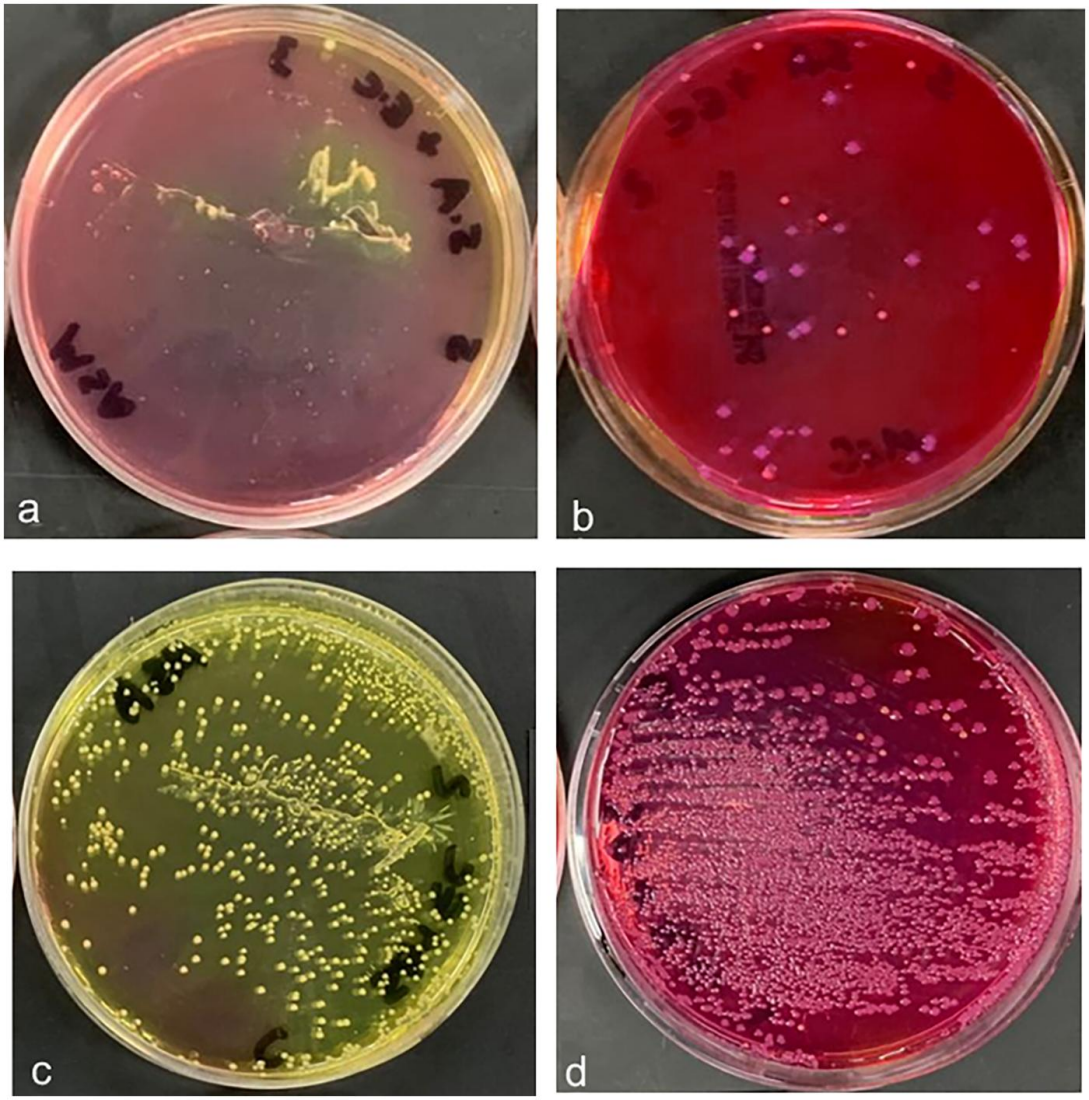
Representative agar plate images showing colony-forming unit (CFU) growth from dual-species (*Staphylococcus aureus* + *Escherichia coli*) biofilms after 24 h of incubation at 37 °C. Upper row (Ti–HA coated discs): **(a)** *S. aureus* colonies on mannitol salt agar (MSA), showing very few colonies; **(b)** *E. coli* colonies on MacConkey agar (MCA), showing sparse colony formation. Lower row (uncoated Ti discs): **(c)** *S. aureus* colonies on MSA, showing dense colony growth; **(d)** *E. coli* colonies on MCA, showing abundant colony formation. The marked difference in colony density between coated **(a,b)** and uncoated **(c,d)** surfaces visually corroborates the quantitative CFU reductions observed for both species in the dual-species biofilm model. Notably, the MSA medium in panel **(a)** retained its original pink color due to insufficient bacterial metabolic activity to alter the phenol red pH indicator, whereas in panel **(c)** the medium turned yellow, confirming extensive mannitol fermentation by the dense S. aureus colonies providing additional visual evidence of the differential bacterial burden between coated and uncoated surfaces.

Plates were incubated for 24 h at 37 °C. Colony-forming units (CFU) were counted and expressed as CFU/cm^2^ of disc surface area for quantitative assessment (Figures 1, 2).

Each titanium disc represents an independent technical replicate (*n* = 8 per group per condition); all discs were prepared from the same material batch, coated under identical sputtering conditions, and inoculated from the same standardized bacterial suspension this constitutes technical replication.

### 2.5 Scanning electron microscopy (SEM) of biofilms

**Figure 3:**
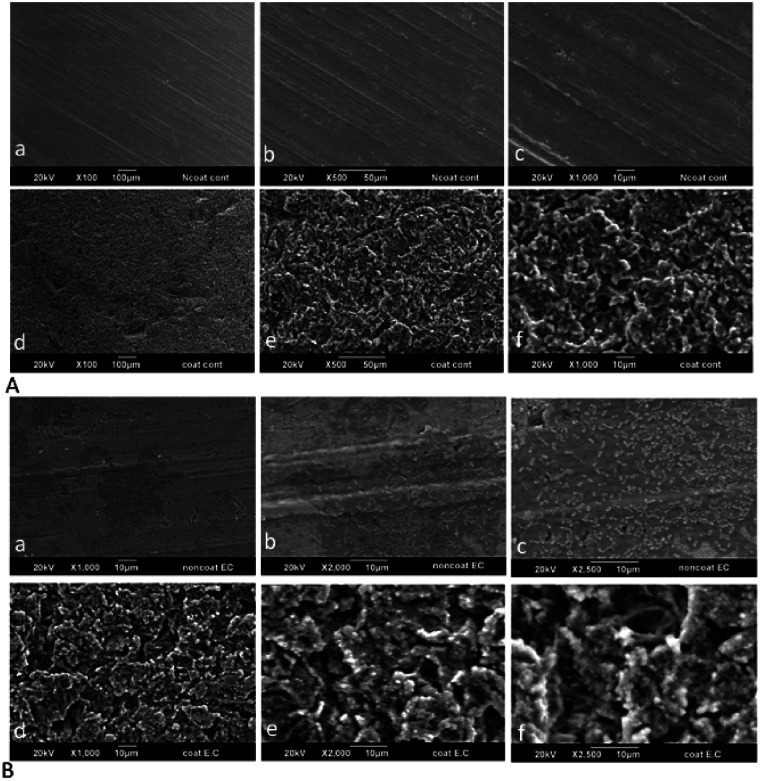
**(A)** Scanning electron micrographs of titanium disc surfaces prior to bacterial inoculation. Upper row: uncoated Ti surfaces at **(a)** ×100, **(b)** ×500, and **(c)** ×1,000 magnification, showing the sandblasted and acid-etched surface topography. Lower row: hydroxyapatite-coated Ti (Ti–HA) surfaces at **(d)** ×100, **(e)** ×500, and **(f)** ×1,000 magnification, demonstrating the characteristic globular morphology of the sputtered HA coating following hydrothermal treatment. Scale bars are ×100 (100 µm), ×500 (50 µm), ×1,000 (10 µm). **(B)** Scanning electron micrographs of *Escherichia coli* biofilm formation on titanium disc surfaces after 24 h of incubation. Upper row: uncoated Ti surfaces at **(a)** ×1,000, **(b)** ×2,000, and **(c)** ×2,500 magnification, showing dense bacterial colonization with rod-shaped *E. coli* cells adhering to the surface. Lower row: Ti–HA surfaces at **(d)** ×1,000, **(e)** ×2,000, and **(f)** ×2,500 magnification, demonstrating visibly reduced bacterial adhesion and biofilm coverage on the HA-coated surface. Scale bars are (10 µm).

**Figure 4:**
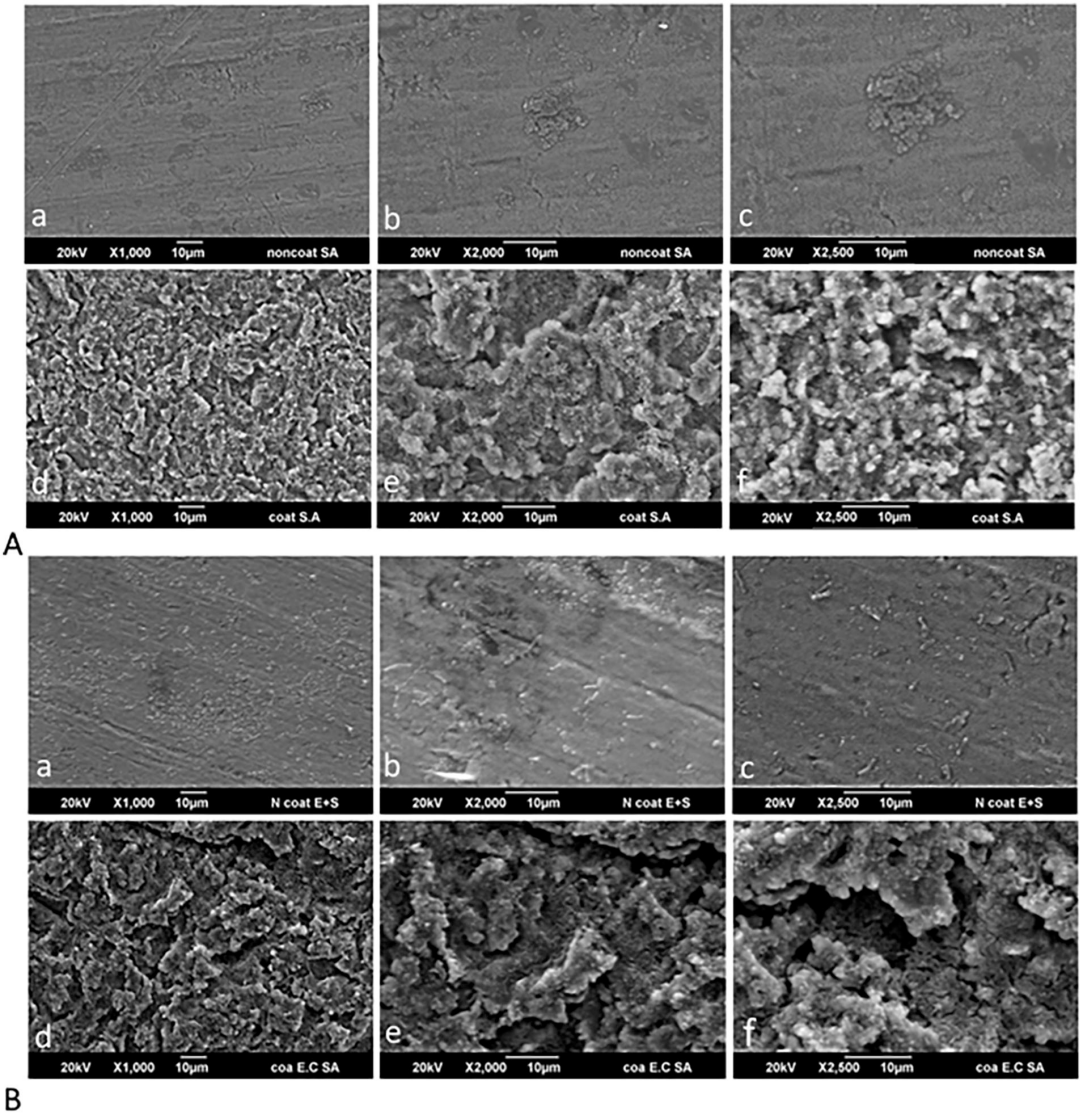
**(A)** Scanning electron micrographs of *Staphylococcus aureus* biofilm formation on titanium disc surfaces after 24 h of incubation. Upper row: uncoated Ti surfaces at **(a)** ×1,000, **(b)** ×2,000, and **(c)** ×2,500 magnification, showing extensive colonization with spherical *S. aureus* cocci arranged in characteristic grape-like clusters adhering to the surface. Lower row: Ti–HA surfaces at **(d)** ×1,000, **(e)** ×2,000, and **(f)** ×2,500 magnification, demonstrating markedly reduced bacterial adhesion and sparse distribution of *S. aureus* cells on the HA-coated surface. Scale bars are indicated on each micrograph (10 µm). **(B)** Scanning electron micrographs of dual-species (*Staphylococcus aureus* and *Escherichia coli*) biofilm formation on titanium disc surfaces after 24 h of incubation. Upper row: uncoated Ti surfaces at **(a)** ×1,000, **(b)** ×2,000, and **(c)** ×2,500 magnification, showing a dense polymicrobial biofilm comprising both spherical cocci (*S. aureus*) and rod-shaped bacilli (*E. coli*) co-colonizing the surface. Lower row: Ti–HA surfaces at **(d)** ×1,000, **(e)** ×2,000, and **(f)** ×2,500 magnification, demonstrating substantially reduced polymicrobial biofilm formation, with visibly fewer bacterial cells of both morphologies on the HA-coated surface. Scale bars are (10 µm).

Qualitative assessment of biofilm morphology was performed using SEM. Pre-washed specimens (*n* = 3 per group) were fixed with 3% glutaraldehyde (Sigma-Aldrich) for 30 min, then dehydrated through a graded ethanol series (30%, 50%, 70%, 90%, and 100%) for 10 min at each concentration. Specimens were subsequently dried using a graded series of hexamethyldisilazane (HMDS; Sigma-Aldrich) solutions for 10 min at each step. Dried samples were mounted on aluminum stubs with double-sided carbon tape and sputter-coated with a conductive gold–palladium film (SPI-Module sputter coater). Biofilm architecture on single-species and dual-species colonized disc surfaces was imaged using a TESCAN VEGA3 LM-SEM (Tescan Orsay Holding, Kohoutovice, Czech Republic) Figures 3, 4.

### 2.6 Sample size estimation

In this study each titanium disc represents one experimental unit. The primary outcome was the number of viable adherent bacteria by counting colony-forming units per square centimeter (CFU/cm^2^) on the different surfaces after 24 h of incubation under three culture conditions: *S. aureus* alone, *E. coli* alone, and dual-species culture. Each titanium disc represents an independent technical replicate (*n* = 8 per group per condition); all discs were prepared from the same material batch, coated under identical sputtering conditions, and inoculated from the same standardized bacterial suspension, constituting technical replication appropriate for controlled *in vitro* surface studies. Sample size estimation was based on detecting a large effect size (Cohen's *d* = 1.2–1.5) between Ti and Ti–HA surfaces, assuming a significance level (*α*) of 0.05 and statistical power (1–*β*) of 0.80. Under these parameters, a minimum of 8 discs per surface type per culture condition was required. Accordingly, the total number of specimens was distributed as follows: for CFU analysis, 48 discs (2 surface types × 3 culture conditions × 8 discs per group); for surface roughness (Ra) measurement, 16 discs (8 per surface type); and for SEM analysis, 18 discs (2 surface types × 3 culture conditions × 3 discs per group). The total number of titanium discs used in the study was 82.

### 2.7 Statistical analysis

CFU values were log-transformed [log_10_(CFU + 1)] to reduce skewness and approximate a normal distribution (28). Normality of the transformed data was assessed using the Shapiro–Wilk test, and homogeneity of variances was evaluated using Levene's test.

A two-way analysis of variance (two-way ANOVA) was performed on the log-transformed CFU data, with surface type (Ti vs. Ti-HA) and culture condition (*S. aureus* single, *E. coli* single, *S. aureus* in mixed culture, and *E. coli* in mixed culture) as fixed factors. Eta-squared (*η*^2^) was calculated to quantify the proportion of total variance explained by each factor. For pairwise comparisons between Ti-HA and uncoated Ti within each culture condition, Mann–Whitney *U* tests were used as a conservative non-parametric complement, because several individual subgroups particularly the Ti-HA conditions with near-zero CFU values did not fully satisfy normality assumptions even after log-transformation. This two-tier analytical strategy, applying a parametric model to the overall dataset while using non-parametric tests for specific subgroup comparisons that violated assumptions, is consistent with established biostatistical guidance for skewed biological count data (29). The percentage reduction in mean CFU was calculated as [(mean Ti − mean Ti–HA)/mean Ti] × 100. Cohen's *d* was calculated on log-transformed data to quantify effect sizes.

To assess whether the antibacterial effect of the HA coating varied across bacterial conditions, within-surface comparisons were performed using the Kruskal–Wallis test. When significant, *post hoc* pairwise comparisons were conducted using Mann–Whitney *U* tests with Bonferroni correction for multiple comparisons.

Biofilm formation was additionally assessed by measuring optical density (OD) at 630 nm using a microplate reader. OD values for Ti-HA and uncoated Ti were compared within each culture condition using Mann–Whitney *U* tests. Ra between the two surface types was compared using an independent-samples *t*-test or Mann–Whitney *U* test, depending on normality.

To evaluate whether surface roughness influenced bacterial adhesion, Spearman correlation analyses were performed between Ra values and CFU counts within each surface group (Ti–HA and uncoated Ti separately), as well as within each individual culture condition. Analyses were conducted within groups rather than across the pooled dataset to avoid confounding attributable to group membership. All statistical analyses were performed using IBM SPSS Statistics version 26.0 (IBM Corp., Armonk, NY, USA) and Python (SciPy v1.11). Data are reported as mean ± standard deviation (SD). Statistical significance was set at *p* < 0.05 for all analyses.

## 3 Results

### 3.1 Surface roughness (Ra value)

The Ra values of the Ti and HA-Ti discs were 1.25 ± 0.26 and 1.13 ± 0.39 µm, respectively.

### 3.2 Viable biofilm quantification (CFU analysis)

Colony-forming unit (CFU) counts recovered from Ti–HA surfaces were dramatically lower than those from uncoated Ti surfaces across all four bacterial conditions (Table 1; Figure 1). For single-species *S. aureus* biofilms, the mean CFU/cm^2^ on Ti–HA was 33.8 ± 24.5 compared to 11,547.5 ± 9,415.4 on uncoated Ti, representing a 99.7% reduction (Mann–Whitney *U* = 0.0; *p* < 0.001). For single-species *E. coli* biofilms, the mean CFU/cm^2^ on Ti–HA was 541.2 ± 258.4 compared to 26,495.0 ± 1,950.1 on uncoated Ti, representing a 98.0% reduction (*U* = 0.0; *p* < 0.001).

**Table 1:** Colony-forming unit counts (CFU/cm^2^) on Ti–HA and uncoated Ti surfaces (*n* = 8 per group).

Culture condition	Ti–HA	Uncoated Ti	*U*	*p*	% Red.	Cohen's *d*
	Mean ± SD	Mean ± SD				
*S. aureus* (single)	33.8 ± 24.5	11,547.5 ± 9,415.4	0.0	<0.001***	99.7%	5.92
*E. coli* (single)	541.2 ± 258.4	26,495.0 ± 1,950.1	0.0	<0.001***	98.0%	10.70
*S. aureus* (mixed)	20.0 ± 16.9	3,837.5 ± 2,408.7	0.0	<0.001***	99.5%	5.44
*E. coli* (mixed)	322.5 ± 55.5	15,290.0 ± 1,157.5	0.0	<0.001***	97.9%	26.81^a^

Mann–Whitney *U* test. % Red., percentage reduction in mean CFU. Cohen's *d* calculated on log_10_(CFU+1)-transformed data.

a Arithmetically verified. The large *d* reflects the very low within-group variance of the Ti–HA condition (322.5 ± 55.5 CFU/cm^2^), producing a very small, pooled SD on the log scale relative to the mean difference (≈1.67 log units). See % CFU reduction (97.9%) for the clinically interpretable effect magnitude.

*** *p* < 0.001.

**Figure 5:**
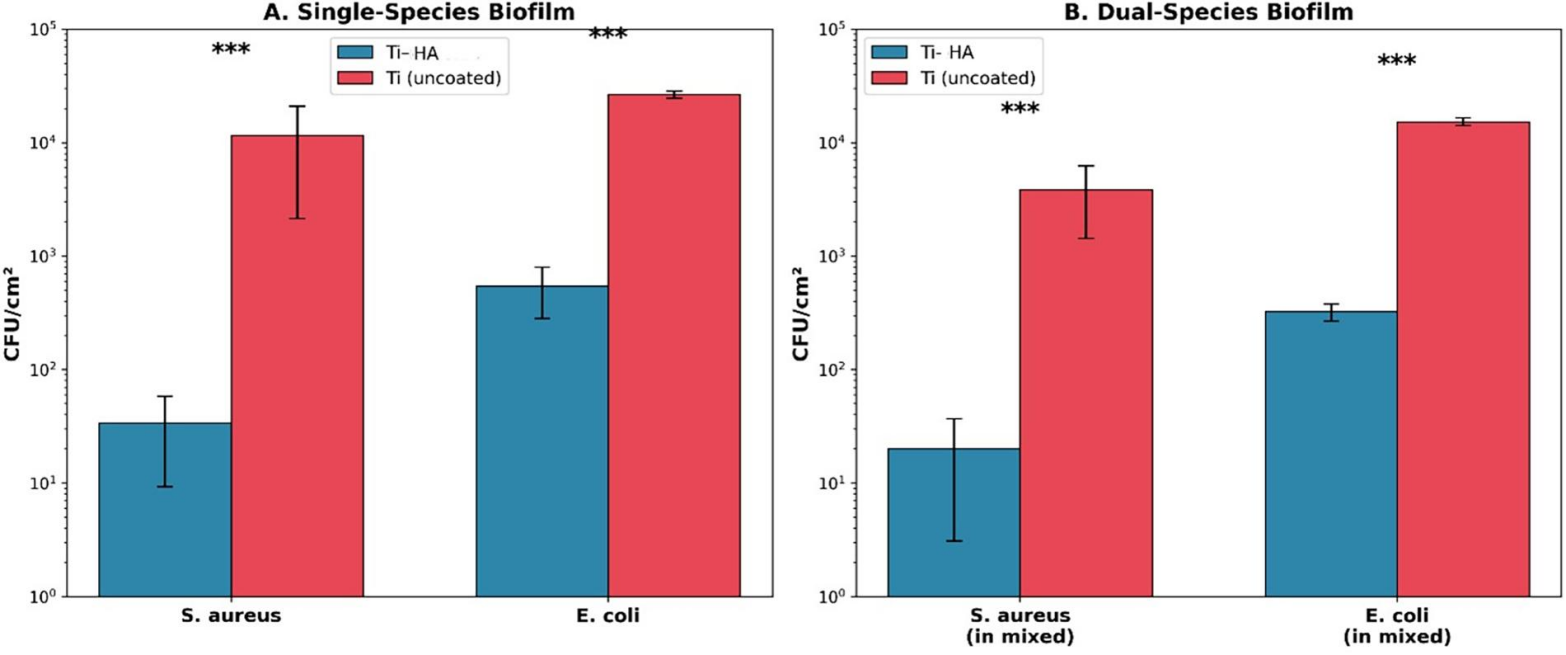
Colony-forming unit counts (CFU/cm^2^, log scale) on Ti–HA and uncoated Ti surfaces. **(A)** Single-species biofilms of *S. aureus* and *E. coli*. **(B)** Dual-species biofilms. Bars represent mean ± SD (*n* = 8). ***p < 0.001 (Mann–Whitney *U* test).

In dual-species biofilms, a similar pattern was observed. The *S. aureus* component recovered from Ti–HA surfaces averaged 20.0 ± 16.9 CFU/cm^2^ compared to 3,837.5 ± 2,408.7 on uncoated Ti, a 99.5% reduction (*U* = 0.0; *p* < 0.001). The *E. coli* component averaged 322.5 ± 55.5 CFU/cm^2^ on Ti–HA vs. 15,290.0 ± 1,157.5 on uncoated Ti, a 97.9% reduction (*U* = 0.0; *p* < 0.001). All comparisons yielded very large effect sizes (Cohen's *d* ranging from 5.44 to 26.81) (Table 1; Figure 5).

**Figure 6:**
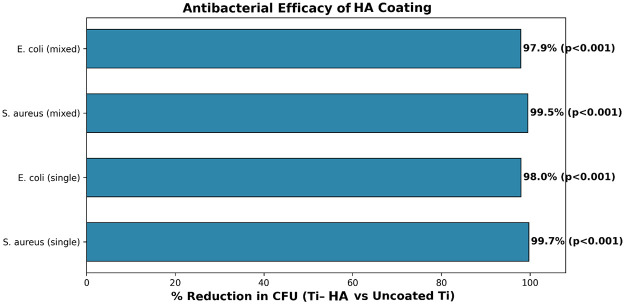
Percentage reduction in mean CFU on Ti–HA relative to uncoated Ti surfaces across single- and dual-species culture conditions. All comparisons ***p < 0.001.

### 3.3 Two-way ANOVA

A two-way ANOVA on log_10_(CFU+1)-transformed data confirmed highly significant main effects for both surface type [*F*_(1, 56)_ = 695.6; *p* < 0.001; *η*^2^ = 0.741] and culture condition [*F*_(3, 56)_ = 55.6; *p* < 0.001; *η*^2^ = 0.178]. A significant surface × culture interaction was also observed [*F*_(3, 56)_ = 6.68; *p* < 0.001; *η*^2^ = 0.021], indicating that the magnitude of the antibacterial effect varied across bacterial conditions (Table 2). The surface type factor accounted for 74.1% of the total variance, confirming that the HA coating was the dominant determinant of bacterial viability.

**Table 2:** Two-way ANOVA results for log_10_(CFU+1)-transformed data.

Source	SS	df	MS	*F*	*p*	*η* ^2^
Surface	67.594	1	67.594	695.6	<0.001***	0.741
Culture condition	16.201	3	5.400	55.6	<0.001***	0.178
Surface × culture	1.948	3	0.649	6.68	<0.001***	0.021
Error	5.442	56	0.097			
Total	91.184	63				

*η*^2^, eta-squared (proportion of total variance).

*** *p* < 0.001.

### 3.4 Biofilm optical density (OD 630 nm)

**Figure 7:**
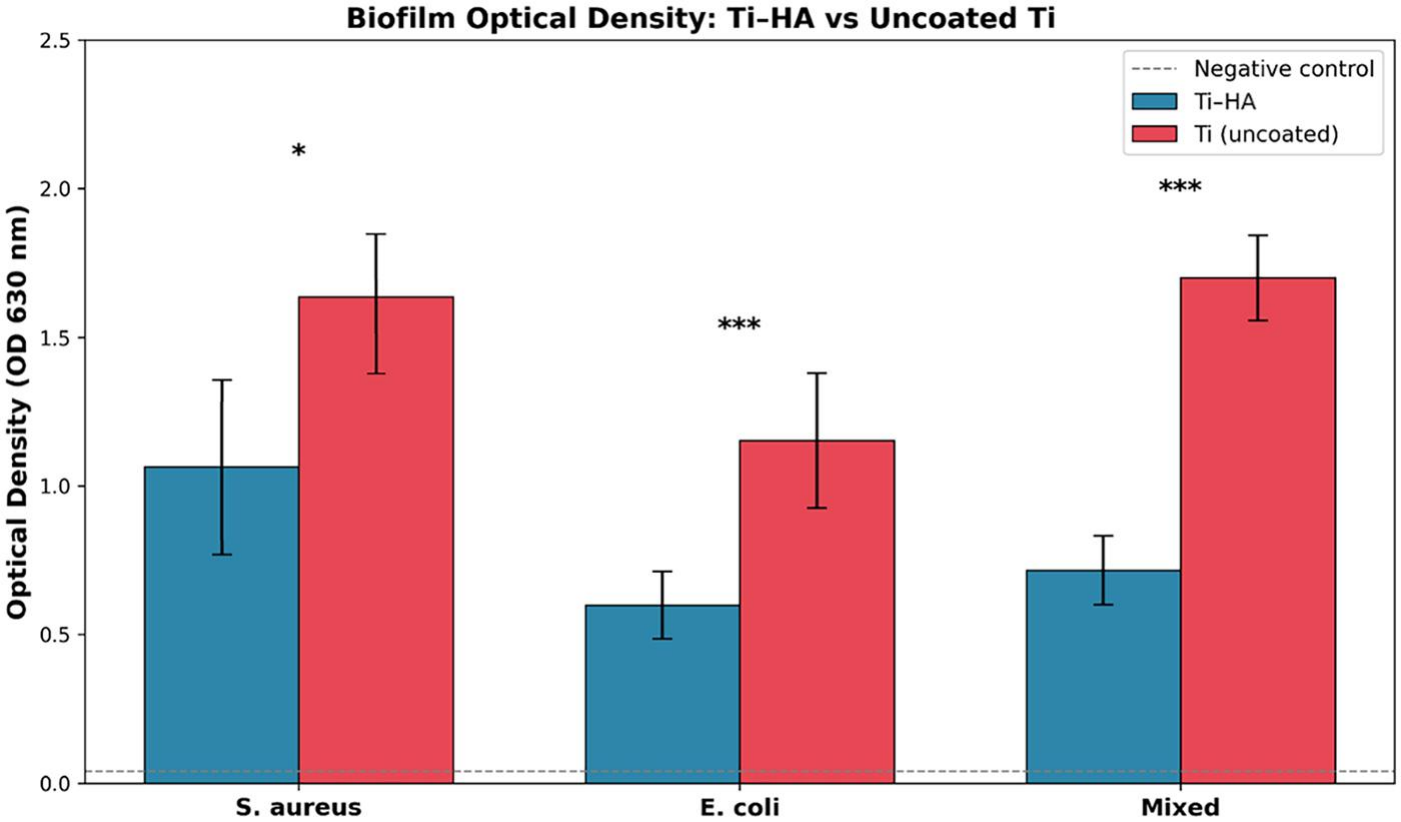
Biofilm optical density (OD 630 nm) on Ti–HA and uncoated Ti surfaces for *S. aureus*, *E. coli*, and mixed-culture conditions. Dashed line indicates negative control (OD = 0.04). Bars represent mean ± SD (*n* = 8). *p < 0.05, ***p < 0.001 (Mann–Whitney *U* test).

OD measurements at 630 nm corroborated the CFU findings, demonstrating significantly lower biofilm density on Ti–HA surfaces across all three culture conditions (Table 3, Figure 7). The greatest difference was observed for the mixed-culture condition (Ti–HA: 0.716 ± 0.115 vs. Ti: 1.700 ± 0.144; *U* = 0.0; *p* < 0.001; Cohen's *d* = 7.56), followed by *E. coli* (0.599 ± 0.113 vs. 1.153 ± 0.227; *U* = 0.0; *p* < 0.001; *d* = 3.09). For *S. aureus*, OD values were significantly lower on Ti–HA (1.065 ± 0.541 vs. 1.636 ± 0.328; *U* = 11.0; *p* = 0.031; *d* = 1.28), although the wider variability in the coated group reflected greater inter-specimen heterogeneity for this species.

**Table 3:** Biofilm optical density (OD 630 nm) on Ti–HA and uncoated Ti surfaces (*n* = 8 per group).

Culture	Ti–HA	Uncoated Ti	*U*	*p*	*d*	Control
	Mean ± SD	Mean ± SD				
*S. aureus*	1.065 ± 0.541	1.636 ± 0.328	11.0	0.031*	1.28	1.63
*E. coli*	0.599 ± 0.113	1.153 ± 0.227	0.0	<0.001***	3.09	1.52
Mixed	0.716 ± 0.115	1.700 ± 0.144	0.0	<0.001***	7.56	1.59

Mann–Whitney *U* test. *d* = Cohen's *d*. Control = positive control OD. Negative control OD = 0.04.

* *p* < 0.05.

*** *p* < 0.001.

The negative control (sterile medium) yielded an OD of 0.04, confirming negligible background absorbance. Positive controls for *S. aureus* (OD = 1.63), *E. coli* (OD = 1.52), and mixed culture (OD = 1.59) confirmed biofilm formation under the experimental conditions.

### 3.5 Differential antibacterial effect by bacterial species

Within the Ti–HA surface, CFU counts differed significantly across culture conditions (Kruskal–Wallis *H* = 24.33; *p* < 0.001). *Post hoc* pairwise comparisons with Bonferroni correction revealed that *S. aureus* single-species (mean: 33.8 CFU/cm^2^) and *S. aureus* in mixed culture (mean: 20.0 CFU/cm^2^) both had significantly lower CFU counts than *E. coli* single-species (mean: 541.2; *p*_adj_ = 0.005) and *E. coli* in mixed culture (mean: 322.5; *p*_adj_ = 0.005). No significant difference was found between *S. aureus* single and mixed conditions (*p*_adj_ = 1.000), or between *E. coli* single and mixed conditions (*p*_adj_ = 0.936). This indicates that the HA coating exhibited a more pronounced antibacterial effect against Gram-positive *S. aureus* compared to Gram-negative *E. coli*, and that the dual-species environment did not significantly alter the antibacterial performance of the coating for either species.

Ti–HA surfaces demonstrated overwhelming antibacterial efficacy compared to uncoated Ti, with 97.9%–99.7% reductions in viable bacterial counts across all single- and dual-species conditions (all *p* < 0.001). This was corroborated by significantly lower biofilm optical densities on Ti–HA surfaces (all *p* ≤ 0.031). Two-way ANOVA confirmed that surface type was the dominant determinant of bacterial viability, accounting for 74.1% of total variance. The antibacterial effect was species-dependent, with greater efficacy against *S. aureus* than *E. coli* (Figure 6).

### 3.6 Correlation analysis between surface roughness (Ra) and bacterial adhesion (CFU/cm^2^)

Spearman correlation analyses performed within each surface group revealed no significant relationship between surface roughness (Ra) and bacterial adhesion (CFU/cm^2^) in either the Ti–HA group (*r*_s_ = - 0.035, *p* = 0.848, *n* = 32) or the uncoated Ti group (*r*_s_ = - 0.201, *p* = 0.269, *n* = 32). Similarly, no significant Ra–CFU correlation was observed within any individual culture condition (all *p* > 0.10). These findings confirm that the marked differences in bacterial adhesion between surface types cannot be attributed to surface roughness variation and are more probably explained by the chemical composition and topographic properties of the HA coating.

## 4 Discussion

The present study demonstrated that hydroxyapatite-coated titanium surfaces produced by RF magnetron sputtering exhibited substantial antibacterial efficacy, with CFU reductions ranging from 97.9% to 99.7% against both *Staphylococcus aureus* and *Escherichia coli* in single-species and dual-species biofilm models. Two-way ANOVA confirmed that surface type was the dominant determinant of bacterial viability, accounting for 74.1% of the total variance. Notably, the antibacterial effect was species-dependent, with greater potency observed against the Gram-positive *S. aureus* than the Gram-negative *E. coli*.

These findings were corroborated by optical density measurements, which demonstrated significantly lower biofilm density on Ti–HA surfaces across all culture conditions, thereby strengthening the overall validity of the results through methodological concordance between quantitative (CFU) and semi-quantitative (OD) assessments. OD at 630 nm is a semi-quantitative measure of total biofilm biomass comprising viable cells, dead bacteria, and extracellular polymeric substances and does not independently confirm bacterial killing (13). OD data were therefore interpreted as a corroborating index of reduced overall biofilm accumulation, with CFU counts serving as the primary measure of bacterial viability.

While higher surface roughness typically encourages bacterial shelter, Micro and nanoscale modification can create anti-adhesive properties. Plasma Electrolytic Oxidation (PEO) increases surface roughness while simultaneously significantly enhancing hydrophilicity, which effectively limits the attachment of common pathogens (30–32).

The higher resistance of Gram-negative *E. coli* compared to Gram-positive *S. aureus* is attributed to the presence of an outer membrane containing lipopolysaccharides (LPS). This outer layer acts as an additional protective barrier against environmental stressors and chemical agents (30). In contrast, *S. aureus* lacks this outer membrane, leaving its thick peptidoglycan layer directly exposed to the HA surface and any antimicrobial agents (e.g., ions or ROS) released from the coating (33).

Despite the protective barrier, Kar et al. (30) demonstrate that—surfaces treated by Plasma Electrolytic Oxidation still achieve substantial reductions in *E. coli* CFUs, indicating that the combined effects of enhanced hydrophilicity and surface energy can overcome Gram-negative defensive mechanisms (34–36). In dual-species models involving *S. aureus* and *E. coli*, researchers found that the antibacterial agents applied to nanohydroxyapatite remained efficient against the polymicrobial community (33). While polymicrobial biofilms are typically more resistant, the observed effectiveness suggests the HA coating may primarily target initial adhesion (37, 38). A 24-hour timeframe may not allow for the full synergistic maturation often seen in chronic infections (37).

HA coatings supplemented with silver nanoparticles have shown 97.5% reduction in biofilm formation and 100% mortality in suspension (13). Which represent a possible alternative to traditional chlorhexidine (CHX) or antibiotic-loaded coatings (33). Antibacterial metal alloys, particularly those incorporating copper (Cu) and silver (Ag), have demonstrated broad-spectrum efficacy against a wide range of pathogens through mechanisms involving elemental alloying and thermal processing. Both Ag and Cu have been extensively documented as potent antibacterial agents, and their incorporation into titanium alloy systems has shown promising results in preventing bacterial adhesion and inhibiting microbial proliferation on implant surfaces (39).

Antimicrobial peptides (AMPs) which are short cationic peptides of 12–50 amino acids have shown to exhibit broad-spectrum activity against both Gram-positive and Gram-negative bacteria primarily through electrostatic disruption of bacterial cell membranes (40). When applied as coatings on titanium and zirconia implant surfaces, AMPs have demonstrated effective inhibition of *Staphylococcus aureus* and *Porphyromonas gingivalis* biofilms while preserving osteoblast adhesion (41, 42). However, further research is going to avoid excessive antibiotic release that may induce cytotoxicity and drug resistance (43).

HA is superior to silver or CHX because it combines antibacterial activity with high biocompatibility and osteoconductivity. It promotes bone-to-implant contact (BIC) measured at up to 98.1% for sputtered HA without the cytotoxicity concerns associated with higher concentrations of metals or disinfectants (13, 24). RF magnetron sputtering creates ultrathin, highly adherent films that exhibit less marginal bone loss under peri-implantitis conditions compared to thick plasma-sprayed coatings, which are prone to delamination (24, 25, 44, 45).

Because *S. aureus* is a strong biofilm producer, the remaining dead bacteria and their substantial extracellular polysaccharide matrix would still contribute to an OD signal even if viability (CFU) is reduced by 99.7% (46). This conflict suggests the HA coating may be bactericidal (killing the cells on contact) rather than purely anti-adhesive. If it were purely anti-adhesive, both CFU and OD would show low values as fewer cells would be present on the surface initially (46).

For *S. aureus* group, the very low CFU observed with a relatively higher OD (1.065 ± 0.541) is consistent with bactericidal mechanism, in which dead bacterial cells and residual extracellular matrix continue to contribute to the OD signal even when viability is reduced.

The demonstrated efficacy exceeding 97% bacterial reduction against both Gram-positive and Gram-negative species, including under polymicrobial conditions, is clinically relevant given the polymicrobial nature of peri-implantitis (30, 37). Similar findings were reported by Rafiei et al. (47) in which they observed that magnesium implants coated with single step dip-coating HA exhibited superior antibacterial performance, against both *S. aureus* and *E. coli* than two-step dip-coating HA coating. The study concluded that both coating strategies improved the surface characteristics of magnesium-based implants, with the two-step HA/Ti coating enhancing corrosion resistance and the single-step HA/Ti coating offering superior antibacterial activity, collectively representing a promising dual-functional approach for biodegradable implant development.

The ultra-thin HA coating achieved via RF magnetron sputtering preserves implant macro-geometry and thread design, facilitating clinical translation without modification to existing implant systems. Thus, implants with a thin sputter-HA coating may offer a promising clinical relevancy by reducing early microbial colonization and potentially lowering the risk of disease onset in susceptible patients. Such surface modifications could contribute to improved long-term implant outcomes by addressing the polymicrobial nature of peri-implant infections (47).

In this study, the 24-hour incubation period used reflects early-stage bacterial adhesion and does not capture the full developmental cycle of mature biofilm. Thus, extended incubation periods (48 h, 72 h, and ≥7 days) are required to evaluate the durability of the antibacterial effect against established, mature biofilms and to assess potential resistance development over time.

Although biocompatibility of sputtered HA coatings has been established in prior studies (24, 25), no cytotoxicity data were generated in the present *in vitro* study. Future work should include cell viability, adhesion, and proliferation assays using human gingival fibroblasts and osteoblasts to confirm that this HA coating with this unique surface topography does not compromise cytocompatibility.

It is important to note that the current findings are exploratory and based on a controlled laboratory environment. While the results demonstrate a significant reduction in bacterial colonization, these *in vitro* observations provide proof-of-concept evidence rather than definitive clinical proof.

The transition from laboratory to clinical application necessitates further evaluation under conditions that more closely mimic the complex oral environment. Factors such as salivary pellicle formation, physiological shear forces from mastication and tongue movement, immune system interactions, and sustained mechanical loading may significantly influence both the coating's durability and its bio-interactive properties. Consequently, extensive *in vivo* modeling is required to substantiate these clinical claims and confirm the long-term efficacy of sputtered HA surfaces in a biological context.

While the current model demonstrates the potent efficacy of sputtered HA coatings against *S. aureus* and *E. coli*, it represents a simplified microbiological environment. In the clinical setting, peri-implantitis is driven by synergistic interactions between different microbial species. These collaborative interactions support the growth of various bacterial species and lead to the formation of structurally complex, mature multi-species biofilms (48).

The absence of late-colonizing anaerobes (e.g., P. gingivalis, Treponema denticola) and the structural protection afforded by a mature extracellular polymeric substance (EPS) matrix may result in different bacterial susceptibility profiles *in vivo* (49). Consequently, while our findings confirm the intrinsic antibacterial potential of the surface, the translation of these results to the oral cavity must be interpreted with caution, as the clinical ecology may modulate the coating's long-term effectiveness.

Surface characterization in this study was limited to morphological (SEM) and roughness (Ra) assessment. Future studies should include x-ray photoelectron spectroscopy (XPS) for elemental composition and Ca/P ratio verification of the deposited coating, Fourier-transform infrared spectroscopy (FTIR) for phase identification, x-ray diffraction (XRD) for phase purity and crystallinity assessment, ROS quantification, time-resolved ion release profiling (Ca^2^⁺, PO_4_^3^⁻), zeta potential and contact angle measurement for wettability, and adhesion strength testing to comprehensively link surface physicochemical properties to antibacterial performance of the HA coating.

A limitation of this study is the *in vitro* design, which does not fully replicate the complex oral environment, including salivary pellicle formation, shear forces, and immune interactions. Furthermore, the 24-hour incubation period evaluates only early-stage adhesion, necessitating extended timelines to assess effects on mature biofilms and long-term durability. Future studies should evaluate the antibacterial performance of HA-coated surfaces against primary oral pathogens and multi-species anaerobic models such as S. mutans, P. gingivalis, and F. nucleatum to better simulate the clinical ecology of peri-implantitis. Additionally, cytotoxicity testing on human gingival fibroblasts and mechanical stability assessments under simulated loading are essential to establish the coating's biocompatibility, durability, and precise mechanism of action.

## 5 Conclusion

Under the conditions of this *in vitro* study, HA-coated titanium surfaces produced by RF magnetron sputtering demonstrated substantial early-stage antibacterial efficacy, achieving 97.9%–99.7% reductions in viable bacterial counts against both Gram-positive *S. aureus* and Gram-negative *E. coli* in single and dual-species biofilm models. These preliminary findings support further investigation of sputtered HA coatings as a potential dual-function implant surface strategy. The clinical relevance of these findings requires confirmation through mature biofilm models, *in vivo* studies, cytotoxicity assessment, and mechanical durability testing.

## Data Availability

The original contributions presented in the study are included in the article/Supplementary Material, further inquiries can be directed to the corresponding author.
